# Polymer Microneedles for Localized Drug Delivery in Musculoskeletal Tissue Regeneration

**DOI:** 10.3390/jfb17070325

**Published:** 2026-07-06

**Authors:** Seihyun Park, Dohee Kim, Hongyoon Kim, Inseon Kim, Seunghun S. Lee

**Affiliations:** Department of Biomedical Engineering, Dongguk University, Seoul 04620, Republic of Korea; parksam0405@naver.com (S.P.);

**Keywords:** polymer microneedle, musculoskeletal regeneration, localized drug delivery, bone, cartilage, stimuli-responsive biomaterial

## Abstract

Musculoskeletal (MSK) disorders—osteoporosis, osteoarthritis, rheumatoid arthritis, intervertebral disc degeneration, tendinopathy, and skeletal muscle injury—contribute the largest share of years lived with disability worldwide. Conventional therapy relies on systemic dosing or repeated intra-articular and peri-tissue injections, which suffer from off-target toxicity, poor lesional bioavailability, and low adherence. Polymer microneedles (MNs)—micron-scale projections of biodegradable, dissolving, hydrogel-forming, or composite polymers—have rapidly matured into a versatile platform for minimally invasive, spatially localized, and temporally programmable delivery of small molecules, biologics, nucleic acids, extracellular vesicles, and cells to MSK tissues. This review synthesizes 2018–2026 advances in polymer MN systems engineered specifically for MSK regeneration. We classify dominant polymer chemistries and MN architectures; map fit-for-purpose across bone, cartilage, joint, intervertebral disc, tendon, and skeletal muscle; and survey “smart” MN designs that exploit reactive oxygen species, pH, mechanical, triboelectric, optogenetic, and ultrasonic triggers. We close with a concise conclusion and forward perspective that identifies the key design levers—hybrid MN–scaffold combination products, stimuli-responsive platforms tuned to the MSK micro-environment, and cell- and EV-loaded formats—most likely to have clinical impact.

## 1. Introduction

Musculoskeletal (MSK) disorders constitute the leading source of years lived with disability and a major driver of healthcare expenditure worldwide, encompassing osteoporotic fractures, osteoarthritis (OA), rheumatoid arthritis (RA), intervertebral disc degeneration (IVDD), sarcopenia, tendinopathy, and traumatic injuries to bone, cartilage, ligaments, and skeletal muscle [1,2]. The biological complexity of these tissues—characterized by hierarchical organization, hypocellularity (in cartilage and intervertebral discs), avascularity, mechanically loaded micro-environments, and tissue-specific cytokine milieus—has frustrated decades of attempts to translate single-modality therapeutics from preclinical models into durable clinical outcomes [1,3]. Even where pharmacological agents exist, their efficacy is constrained by three persistent bottlenecks: (i) the systemic dilution and off-target toxicity associated with oral or intravenous administration; (ii) the limited residence time and the inflammatory rebound that follow intra-articular bolus injections of high-molecular-weight biologics such as hyaluronic acid (HA) or anti-cytokine antibodies; and (iii) poor patient adherence to chronic regimens, particularly when dosing requires either professional injection or daily self-administration of subcutaneous biologics [4,5].

Tissue-engineering strategies have addressed parts of this problem by integrating drug-loaded scaffolds, hydrogels, and three-dimensional (3D)-printed implants that locally release growth factors, anti-inflammatory drugs, or cells over timescales of weeks to months [1,2,6]. Hybrid macroporous scaffolds that simultaneously support bone in-growth and sustained drug release [3], and double cryogel platforms that sequentially deliver SDF-1 and BMP-2 to enhance calvarial regeneration [4], illustrate how rationally engineered polymer carriers can recapitulate the kinetics of the natural healing cascade. However, these implant-based approaches typically require open or arthroscopic surgery, are difficult to retrieve or to dose-adjust in situ, and remain less attractive for indications such as OA flare management or peri-articular tendinopathy, where minimal invasiveness is paramount.

Microneedles (MNs)—arrays of micron-scale projections (typically 50–1500 µm in length) that mechanically pierce the stratum corneum or other tissue barriers without engaging dermal nociceptors—were originally conceived as a transdermal alternative to hypodermic needles for vaccines and small molecules [5,6]. Over the past decade, MN technology has moved well beyond cosmetic and vaccine applications; hundreds of designs now exploit polymer chemistries with controllable swelling, dissolution, biodegradation, and stimulus-responsive behavior to deliver biologics, nucleic acids, extracellular vesicles (EVs), and even living cells [7,8,9,10]. Polymer MNs are particularly well suited to MSK indications because (i) the target tissues—bone surfaces overlaid by skin or periosteum, joint capsules accessible peri-articularly, and superficial tendons and muscles—lie within a few millimeters of the skin or mucosal surface, which the longer-needle (≥500 µm) variants of MNs can readily reach; (ii) the slow turnover of MSK tissues benefits from sustained or stimulus-gated release that matches healing timescales of weeks to months; and (iii) the polymer matrices used in MNs (PLGA, poly(vinyl alcohol) (PVA), poly(vinyl pyrrolidone) (PVP), HA, gelatin methacryloyl (GelMA), silk fibroin, and chitosan) overlap substantially with those validated in bone and cartilage tissue-engineering scaffolds, accelerating regulatory familiarity [3,11,12,13,14].

A series of bibliometric analyses have charted the explosive growth of MN research, with the number of MN papers exceeding 1000 per year by 2020, and patent filings, clinical trial registrations, and online-search activity all rising in parallel [11]. Despite this momentum, the published literature is dominated by transdermal applications in vaccinology, dermatology, and diabetes; MSK-specific MN designs have only recently coalesced into a coherent subfield. Existing MN reviews focus predominantly on fabrication techniques [12,13], material classes [10,14], or single indications such as RA [15,16]. To our knowledge, no review has yet synthesized polymer MN delivery across the full MSK spectrum—bone, cartilage, joint, intervertebral disc, tendon, and skeletal muscle—and explicitly mapped polymer chemistry and MN architecture onto indication-specific microenvironment constraints. The opportunity to do so is timely; between 2023 and 2026 alone, several landmark studies have demonstrated MN-mediated delivery of CAR-M-like macrophages for IVDD [17], triboelectric MN patches releasing optogenetically engineered EVs [18], denosumab-loaded dissolving MNs for OA [19], and silk-fibroin MN-LPS-primed BMSC-exosome platforms for oral mucosal regeneration [20].

This review pursues four objectives. First, we provide a structured taxonomy of polymer MN architectures and the polymer chemistries that dominate MSK applications, and we relate this taxonomy to the mechanical, dissolution, and release performance windows that MSK tissues demand. Second, we summarize the MSK microenvironmental constraints—including barrier anatomy, cell density, oxygen tension, mechanical loading, and inflammatory state—that gate MN design choices. Third, we critically map MN-based delivery against the principal MSK indications, drawing connections between architecture, payload, and outcome. Fourth, we appraise emerging stimuli-responsive and “smart” MN systems ).

**Figure 1:**
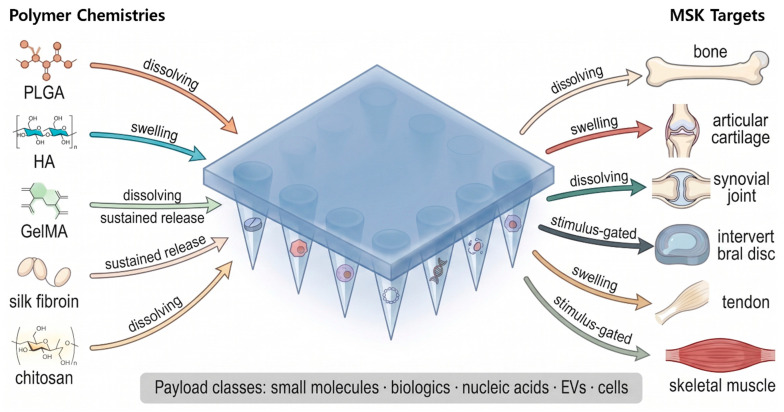
Conceptual overview of polymer microneedle-mediated drug delivery for musculoskeletal tissue regeneration. Central microneedle (MN) patch delivers diverse payload classes (small molecules, biologics, nucleic acids, extracellular vesicles, and cells) to six principal MSK targets—bone, articular cartilage, synovial joint, intervertebral disc, tendon, and skeletal muscle—from a common polymer chemistry palette (PLGA, hyaluronic acid (HA), gelatin methacryloyl (GelMA), silk fibroin, and chitosan). Coupling arrows indicate the dominant release modalities (dissolving, swelling, sustained release, and stimulus-gated). Abbreviations: EV, extracellular vesicle; MN, microneedle; MSK, musculoskeletal; PLGA, poly(lactic-*co*-glycolic acid).

The central thesis is that polymer MNs, when designed with explicit attention to MSK tissue mechanics and pharmacokinetic timescales, can fill a real clinical gap by delivering drugs, biologics, EVs, and cells in a minimally invasive, patient-administrable, dose-titratable fashion (Figure 1) and that this will be achieved most efficiently by hybridizing MNs with the validated polymer-scaffold platforms that the MSK regenerative medicine community has developed over the past two decades [1,4,6,21,22,23].

Scope and literature search: This is a structured narrative (not a systematic PRISMA) review. We searched PubMed, Scopus and Web of Science for English-language records published between January 2018 and the first quarter of 2026, combining the terms (“microneedle” OR “microneedle array” OR “dissolving microneedle” OR “hydrogel microneedle”) with musculoskeletal terms (“bone” OR “osteoporosis” OR “osteoarthritis” OR “cartilage” OR “rheumatoid arthritis” OR “intervertebral disc” OR “tendon” OR “skeletal muscle” OR “periodontal”) and with delivery/stimulus terms (“drug delivery” OR “extracellular vesicle” OR “stimuli-responsive”). Records were screened at the title/abstract level and retained when a polymer microneedle was applied to, or explicitly proposed for, a musculoskeletal indication; purely transdermal cosmetic, vaccine and diabetes-only studies were excluded unless they established an architecture or polymer chemistry transferable to musculoskeletal use. Reference lists of retrieved articles and recent reviews were hand-searched to capture additional primary studies, and landmark mechanistic papers outside the 2018–2026 window were retained where needed for context. Because the field is young and heterogeneous, we present a thematic synthesis rather than a quantitative meta-analysis.

## 2. Polymer Microneedle Architectures and Materials

### 2.1. Architectural Classification

**Figure 2:**
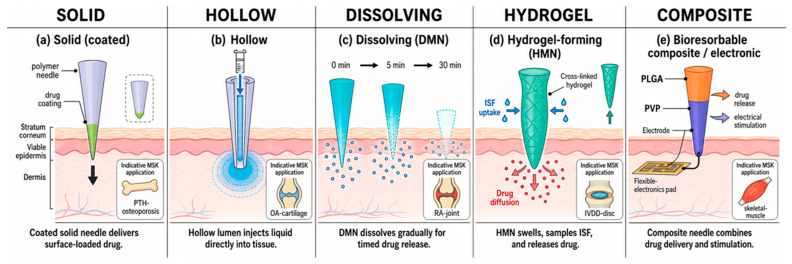
Taxonomy of five canonical polymer microneedle architectures used in MSK applications. (**a**) Solid (coated) MNs: a stiff polymer (or metal) needle core with a drug-bearing coating that dissolves in the interstitial fluid; indicative application: transdermal PTH delivery for osteoporosis [25]. (**b**) Hollow MNs: tubular needles with a lumen for liquid bolus delivery; indicative application: intra-articular biologic dosing. (**c**) Dissolving MNs (DMNs): wholly water-soluble polymers (HA, PVP, PVA, and chitosan) that dissolve within minutes to hours; indicative application: OA denosumab [18] and multi-stage lubricating MNs [36]. (**d**) Hydrogel-forming MNs (HMNs): cross-linked swellable polymers that draw in interstitial fluid [16]. (**e**) Bioresorbable composite/electronic MNs: layered designs combining a fast-release tip with a load-bearing or electrically active backbone; indicative application: IVD triboelectric-responsive EV delivery [18] and skeletal-muscle bioresorbable electronic stimulation [34]. Abbreviations: DMN, dissolving microneedle; HA, hyaluronic acid; HMN, hydrogel-forming microneedle; ISF, interstitial fluid; IVD, intervertebral disc; MN, microneedle; OA, osteoarthritis; PTH, parathyroid hormone; PVA, poly(vinyl alcohol); PVP, poly(vinyl pyrrolidone); RA, rheumatoid arthritis.

Polymer MN designs fall into five canonical architectures (Figure 2) [10,12,13]:

(i) Solid (mechanical disruption) MNs mechanically perforate the barrier and are then withdrawn; the drug is applied either as a topical formulation that follows the micropores (“poke-and-patch”) or as a coating that dissolves in the interstitial fluid (“coat-and-poke”). PLGA, PLA, and PCL solid MNs offer the highest mechanical strength but limited payload capacity and require waste handling [24]. Coated solid MNs were used for some of the earliest MSK applications, including a parathyroid hormone (PTH 1-34)-coated stainless-steel/polymer hybrid system that completed a phase II clinical study for postmenopausal osteoporosis with pharmacokinetics comparable to subcutaneous injection [25].
(ii) Hollow MNs convey a liquid bolus through a microscale lumen, mimicking conventional needles at a smaller scale. Although mostly fabricated from silicon or metal, polymer-only hollow MNs have been realized using 2PP-printed photopolymers and offer attractive control over flow rate and depth [26].
(iii) Dissolving MNs (DMNs) are wholly composed of water-soluble polymers—HA, PVP, PVA, dextran, sucrose, sodium carboxymethyl cellulose, or chitosan—that dissolve in interstitial fluid within minutes to hours, releasing the entrapped drug and leaving no waste sharps [10]. DMNs dominate the MSK literature because of their patient-friendliness, scalability, and compatibility with thermosensitive biologics. Recent examples include teriparatide-loaded self-dissolving HA MNs for osteoporosis [27,28], denosumab-loaded DMNs that suppress macrophage senescence in canine OA [19], and a Phaseolus-polysaccharide DMN that co-delivers sinomenine for RA management [29].
(iv) Hydrogel-forming (swellable) MNs (HMNs) are made from cross-linked polymers (poly(methyl vinyl ether-*co*-maleic acid)/PEG, GelMA, methacrylated HA, sodium alginate, and gellan gum) that swell upon insertion, draw interstitial fluid, and release payload through the swollen matrix [13,30]. Because HMNs remain physically intact, they can be removed in one piece, an advantage for sterility audits and for repeat dosing. HMN designs have proliferated in OA and RA applications, where the swollen network can act as a sustained local depot in close apposition to the synovial membrane [31,32,33].
(v) Bioresorbable composite or hybrid MNs combine a fast-dissolving “tip” loaded with bioactive cargo and a structural “backbone” made of stiffer polymers or even bioresorbable electronics for combined drug and electrical stimulation [34]. Lyophilized PLGA/PVP composite MNs reinforced with biocompatible glue have recently shown 4–7-fold improvements in compressive failure load relative to standard DMNs while retaining the same dissolution kinetics [35].

### 2.2. Polymer Chemistries and Structure–Performance Relations

The choice of polymer governs three coupled properties that determine MSK performance: the mechanical strength of the projection (must exceed the ~0.05–0.1 N/needle threshold for skin and ~0.4 N/needle for joint capsule penetration), the dissolution or degradation rate (sets the temporal release profile), and biocompatibility/biodegradability (sets the safety and immunogenic profile) [12,13,37].

Synthetic polymers. PLGA dominates the sustained-release MN literature because its lactide:glycolide ratio tunes degradation between weeks and months, matching MSK healing timescales. PLGA MNs have been used to deliver carfilzomib for sustained peptide release [38] and curcumin via co-axial electrosprayed mesoporous silica coatings [39]. PVP and PVA are the workhorses for fast-dissolving DMNs because of their low cost, regulatory familiarity, and rapid water solubility; both are widely used in OA-targeted DMNs [40,41]. PEG and PEGDA are used as crosslinkers to tune swelling and moduli, particularly in HMN systems [31].

Natural polymers. HA is the most prevalent natural polymer in MSK MN designs because it is itself the principal lubricant of synovial fluid; HA-based DMNs have been used for naringin delivery in RA and dual-functional melanoma/skin regeneration platforms [31,42]. GelMA and methacrylated silk fibroin (SilMA) provide photo-crosslinkable, ECM-mimicking matrices ideal for cell- or EV-loaded MNs; recent GelMA-SilMA biomimetic dual-crosslinked MNs delivered TAT-modified quercetin liposomes via the caspase-8/3/GSDME pathway in RA [32], while DLP-printed GelMA/SilMA personalized MNs accelerated PRP-mediated wound repair [43]. Silk fibroin alone, with its tunable β-sheet content, supports semi-interpenetrating-network smart MNs [44] and has been used to load LPS-primed BMSC-derived exosomes for oral ulcer treatment [20]. Chitosan, recombinant collagen [45], and fucoidan [46] add complementary chemistries with intrinsic anti-inflammatory or anti-microbial activity. The same polymer families have driven major advances in MSK scaffold engineering—for example, GelMA–bioglass cryogels for bone regeneration [21], heparin-functionalized injectable cryogels for neovascularization [23], and bioceramic-mediated chondrocyte hypertrophy strategies for osteochondral repair [22]—providing a natural translation pathway for polymer choices to migrate from scaffolds into MN formats.

A direct consequence of this material overlap is that polymer chemistries already validated as “regulatory-friendly” in MSK scaffold form (e.g., methacrylated gelatin in cryogels [21], heparin-functionalized hydrogels [23], and silicon nitride composites [36,47,48]) carry forward much of their toxicology, biocompatibility, and degradation data when transposed into MN format, accelerating the formal pharmaceutical development process. The corollary is that the choice of MN polymer for a given MSK indication should be informed not only by mechanical and dissolution targets but also by the existing regulatory dossier of that polymer in nearby products.

Composite and stimuli-responsive systems. Hybrid composites pair structural polymers with bioactive ceramics (whitlockite, β-TCP, hydroxyapatite, and silicon nitride) [49,50,51,52] to add osteogenic or antibacterial functions; this is conceptually identical to the design philosophy that has driven hybrid macroporous scaffolds [3] and silicon nitride cryogels with anti-biofilm and osteogenic effects [52]. Stimuli-responsive polymers (pH-, ROS-, glucose-, NIR-, ultrasound-, mechanical-, or triboelectric-responsive) form the basis of “smart” MN designs discussed in Section 5 [47,48,53,54,55].

## 3. The Musculoskeletal Microenvironment and MN Design Constraints

### 3.1. Anatomical Access and Barrier Considerations

**Figure 3:**
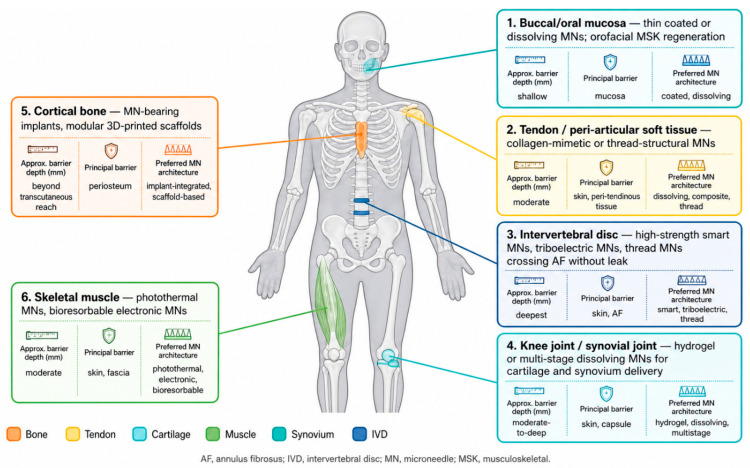
Anatomical access and design constraint map for polymer microneedle delivery across musculoskeletal targets. Callouts summarize, for each MSK compartment, the approximate barrier depth (mm), the principal tissue barrier (skin, joint capsule, annulus fibrosus (AF), or periosteum), and the preferred MN architecture. Buccal/oral mucosa is accessible by thin-coated or dissolving MNs [59] and is a useful proving ground for orofacial MSK regeneration [60]. Tendon and peri-articular soft tissues are reached by collagen-mimetic or thread-structured MNs [45]. The intervertebral disc is the least accessible MSK target and demands high-strength smart MNs [56], triboelectric MNs [18], or thread MNs that cross the AF without leak [57]. The knee joint and other synovial joints are reachable by hydrogel or multi-stage dissolving MNs for cartilage and synovium delivery [15,18,36]. Cortical bone lies beyond transcutaneous MN reach and is reached by MN-bearing implants and modular 3D-printed scaffolds [6,61]. Skeletal muscle is reached by photothermal and bioresorbable electronic MN platforms [34,58].

MSK targets sit at variable depths from the body surface and behind variable barriers (Figure 3). (i) The diaphyseal cortex of long bones lies beneath 2–10 mm of soft tissue and periosteum, generally beyond the reach of skin-applied MNs but accessible peri-operatively to MN-bearing implants. (ii) Joint capsules—knee, finger, wrist, and temporomandibular—are typically 1–3 mm deep and within reach of 800–1500 µm MNs applied to peri-articular skin [36]. (iii) The intervertebral disc requires either fluoroscopy-guided percutaneous access or laminectomy exposure; novel high-strength smart MNs and thread-structured MNs have been engineered specifically to penetrate the annulus fibrosus and deliver payload to the nucleus pulposus [17,18,56,57]. (iv) Tendon and superficial muscle are within 2–5 mm of the skin and have been targeted with carbonized photothermal MN patches for skeletal muscle damage [58] and bioresorbable electronic MNs for combined electrotherapy and drug delivery [34]. (v) Mucosal MSK adjacent tissues (alveolar bone and periodontal ligament) are accessible by buccal MN patches [59,60].

### 3.2. Pharmacokinetic and Microenvironmental Constraints

The MSK micro-environment differs sharply from that of skin or muscle in three regenerative-medicine-relevant ways. (i) Mechanical loading: bone, cartilage, tendon, and IVD experience cyclic loads of 1–10 MPa under physiological activity, which rapidly disperse low-viscosity injectables and necessitate either depot-forming carriers or load-tolerant MN matrices. (ii) Vascularity: cartilage, IVD nucleus pulposus, and the inner meniscus are largely avascular, so systemic small molecules diffuse poorly while locally injected biologics escape rapidly into the synovial circulation; sustained-release MN depots seated at the cartilage–synovium interface bypass this limitation [31,62]. (iii) Inflammatory and oxidative milieu: OA, RA, IVDD, and bone non-union are all characterized by elevated reactive oxygen species (ROS), pro-inflammatory cytokines (IL-1β, IL-6, and TNF-α), matrix metalloproteinases, and (in IVD and aging cartilage) acidic pH due to anaerobic metabolism. These features can be exploited as triggers for stimuli-responsive MNs [53,54,63] but can also degrade payloads such as proteins and EVs that must be protected by the MN polymer matrix.

### 3.3. Cellular Targets and Dosing Windows

The biological logic of MSK delivery often demands sequential or temporally distinct cues, for example, an early angiogenic/anti-inflammatory phase followed by an osteogenic or chondrogenic phase. Lee et al. showed in a calvarial defect model that sequential SDF-1 and BMP-2 release from a double cryogel system enhanced bone formation relative to either factor alone [4], and Kim et al. showed that VEGF-overexpressed adipose-derived stem cells combined with whitlockite-reinforced cryogels improved both vascularization and bone regeneration [49]. MN platforms can recapitulate these multistage profiles either by stratifying polymer layers within a single MN (e.g., a fast-dissolving HA tip releasing the early-phase factor and a slowly degrading PLGA backbone releasing the late-phase factor) [35,64] or by sequential application of two MN patches.

## 4. MN-Mediated Drug Delivery for Musculoskeletal Indications

### 4.1. Bone Regeneration, Osteoporosis, and Bone Metastasis

MN drug delivery has now been demonstrated across the musculoskeletal disease spectrum (Table 1); the longest clinical track record is in osteoporosis. A teriparatide-coated titanium/polymer MN patch (the ZP-PTH system) was evaluated in a randomized, open-label study against subcutaneous teriparatide and showed comparable PTH(1-34) Cmax and AUC profiles, validating the MN route for systemic delivery of an MSK-relevant biologic [25]. Self-dissolving HA MN arrays were subsequently shown to enhance transdermal absorption of recombinant human PTH(1-34) in rats [27], and in 2022, a teriparatide DMN system demonstrated dose-dependent pharmacokinetics that support flexible regimen design [28]. The 2017 first-in-class approval of abaloparatide established the regulatory and commercial appetite for parathyroid-axis bone-anabolic biologics [65], strengthening the case for patient-administrable MN dosage forms [66,67,68,69].

For local bone formation, MN-delivered growth factors and gene therapy vectors are now competitive with implantable scaffolds. Sequential SDF-1/BMP-2 delivery from cryogels potentiated calvarial bone formation [4], and the same logic—early chemotaxis followed by osteoinduction—has been adapted to layered MN designs [35]. A 2024 chitosan/silk-fibroin MN loaded with LPS-pretreated BMSC-derived exosomes accelerated oral ulcer healing, including alveolar epithelial regeneration, indicating the feasibility of EV-based bone-adjacent regenerative therapy [20]. For infection-complicated bone defects, recombinant collagen MNs delivering antibacterial copper–DNA nanoparticles to skin and soft-tissue infections [45] and silicon-nitride-reinforced cryogel platforms with anti-biofilm activity [52] illustrate complementary strategies that MN-based delivery can borrow from established scaffold approaches.

For osteoporotic and metastatic vertebral lesions, the same anatomical accessibility constraints that limit cortical-bone MN applications also point toward MN-bearing implants and 3D-printed scaffolds rather than transcutaneous MN patches; the recent emergence of 3D-printed assemblable bespoke scaffolds and LEGO-inspired titanium scaffolds that incorporate microcryogel modules [61] suggests a hybrid pathway in which MN-style microreservoirs are integrated into modular load-bearing implants. Such hybrid constructs would extend the reach of MN-formatted dosing into the deep MSK compartments that transcutaneous MNs cannot access while inheriting the patient-specific design freedom that 3D-printed scaffolds already enable.

From a head-to-head comparison perspective, transdermal MN delivery of bone-anabolic biologics also offers a meaningful logistical advantage over current standard-of-care subcutaneous dosing. Daily subcutaneous teriparatide is associated with poor 12-month adherence (~40–60% in real-world cohorts), and patient-applied MN patches have been hypothesized to improve adherence by halving the cognitive and pain burden of administration [11,25,28]. A recent in vitro and in vivo comparison of cellular and osteogenic responses across alternative biomaterials for spinal implants further demonstrated that polymer/ceramic combinations selected for MN backings can be tuned to support both bone–material integration and antibacterial activity [55], and triply periodic minimal surface (TPMS) PEEK/silicon nitride scaffolds with optimized strut geometry [47] establish a design space in which MN microreservoirs could be embedded into the strut walls without compromising mechanical performance. A critical caveat tempers this momentum: most bone and osteoporosis MN reports stop at pharmacokinetic bioequivalence or short-term marker changes and seldom report functional endpoints such as bone-mineral-density gain, fracture-healing biomechanics, or callus quality, and almost none verify needle integrity or release fidelity under the cyclic loading of bone, so the translational gap between transdermal PTH delivery and load-bearing repair remains largely uncharacterized.

### 4.2. Osteoarthritis and Cartilage Regeneration

OA is the highest-volume MSK indication and the most active target for MN research [15]. The intra-articular delivery problem is acute: hyaluronate viscosupplementation is rapidly cleared (synovial half-life of HA < 24 h), and corticosteroid injections, while effective short-term, accelerate cartilage thinning in the long term. MN strategies seek to deliver controlled-release depots either intra-articularly or peri-articularly through the joint capsule [70].

Key recent designs include (i) lubricating MN systems with multistage sustained release that simultaneously reduce friction at the chondral surface and release anti-inflammatory drugs, demonstrated in a rat MIA model with significant OARSI score reductions over 8 weeks [36]; (ii) liposome-loaded dissolving MNs that improve the intra-articular delivery of triptolide and reduce systemic toxicity [62]; (iii) PDA-Exo MNs that combine polydopamine-coated stem-cell-derived exosomes with ROS-scavenging polymer matrices to drive PI3K-Akt-mTOR-mediated chondroprotection [31]; (iv) denosumab-loaded DMNs that target macrophage senescence in canine and rodent OA models, providing a translational dataset in a large-animal model [19]; and (v) polysaccharide MNs loaded with *Cucumaria frondosa* polysaccharides plus 3-acetylaconitine for combined analgesic, anti-inflammatory, and chondroprotective activity [71]. Multi-component glycosaminoglycan/chondroitin sulfate/HA-loaded PVP-PEG MNs further demonstrate that the constituent ECM molecules of cartilage can themselves be delivered via MN [41], aligning with bioceramic-mediated chondrocyte-hypertrophy strategies that promote calcified cartilage formation in osteochondral defects [22]. Microemulsion-incorporated DMNs co-delivering celecoxib and α-linolenic acid further illustrate how MN platforms can carry hydrophobic NSAID combinations that are otherwise difficult to formulate for intra-articular use [72]. A 2024 polymeric MN delivering processed human placental tissue showed dual chondroprotective and immunomodulatory effects in a surgical OA model [42].

A common theme across these OA studies is the layered or multi-stage release rationale, which has clear parallels in the staged growth-factor-release strategies developed for bone defect cryogels [4]. The lubricating MN system [36], for example, achieves an early “lubricant burst” mimicking the role of synovial HA, followed by a sustained anti-inflammatory payload, and several DMNs explicitly stratify hydrophilic and hydrophobic cargos into separate polymer layers [72,73]. Mechanistically, OA MN designs increasingly couple cartilage-protective payload delivery with subchondral-bone modulation, recognizing that OA is a whole-joint disease; the denosumab DMN that targets macrophage senescence [19] and the PDA@Exo MN that rebalances anabolic and catabolic processes [31] both engage subchondral bone alongside the cartilage compartment. The chondroprotective polysaccharide MN system [71] additionally introduces analgesia as an explicit design objective, addressing the pain dimension of OA that bench-side cartilage repair models often overlook. The chondrogenic-priming logic developed in bioceramic scaffold systems for osteochondral repair [22] could, in principle, be transposed into MN tip layers, in which a calcium-phosphate- or bioglass-doped tip primes hypertrophic transition, while a deeper polymer layer delivers chondroprotective cargo to the cartilage interface.

#### In-Depth Comparison of OA Microneedle Platforms: Fabrication, Design Logic, and Outcomes

Because OA is the most evidence-dense MSK indication for MN delivery, it is the natural locus for an in-depth, comparative appraisal of how fabrication and architecture map onto therapeutic requirements. The reported OA platforms fall into four fabrication classes that embody distinct design logics. Dissolving microneedles (DMNs)—the liposome-triptolide DMN, the denosumab DMN, the polysaccharide DMN, and the microemulsion celecoxib/alpha-linolenic-acid DMN—dissolve cargo-laden tips in situ and suit single-bolus or short sustained delivery, their principal design variable being the tip polymer and any layered partitioning of hydrophilic versus hydrophobic cargo. Matrix-active systems—the PDA-exosome MN with a ROS-scavenging matrix and the glycosaminoglycan/chondroitin-sulfate/HA PVP-PEG MN—use the polymer matrix itself as a bioactive carrier rather than an inert vehicle. Surface-engineered lubricating MNs add a friction-reducing chondral surface function on top of drug release. A reviewer-relevant gap is that the quantitative fabrication parameters needed to make these classes directly comparable—needle height, tip density, aspect ratio, insertion force, and measured mechanical strength—are reported inconsistently or not at all, so cross-platform design comparison cannot yet be placed on a common quantitative footing (an evidence-reporting gap, not a claim that the data do not exist).

Mapping release strategy onto OA’s biphasic pathology—an early inflammatory phase followed by a chondro-anabolic repair phase—clarifies why layered and stimulus-responsive designs recur. The lubricating multi-stage MN couples an early lubricant burst that substitutes for rapidly cleared synovial HA (synovial half-life under 24 h) with a sustained anti-inflammatory payload, explicitly matching the two phases; PLGA-based sustained release extends delivery to roughly two to four weeks but lacks on-demand control; ROS-responsive matrices release preferentially in the oxidative OA microenvironment, in principle concentrating dose where pathology is active; and liposome- or microemulsion-based DMNs address formulation rather than kinetics, solubilizing hydrophobic NSAIDs and natural products that are otherwise difficult to deliver intra-articularly. The corresponding efficacy readouts, however, are drawn from non-uniform models and timescales—chemical MIA versus surgical destabilization, with a follow-up rarely beyond eight weeks—and outcome metrics (OARSI score, pain behavior, and biomarker shifts) are reported on incommensurable scales. The denosumab DMN supplies the field’s principal large-animal (canine) datapoint; the remainder are small-rodent studies.

Based on current evidence, the best-supported design class for OA is the layered or multi-stage MN that decouples an early anti-inflammatory or lubricating action from a later chondroprotective payload, since this is the feature shared by the platforms reporting the most durable functional benefit (the lubricating multi-stage MN and the denosumab DMN). This is a synthesis of heterogeneous studies rather than a conclusion from head-to-head comparison, and three deficits must be closed before any platform can be ranked as clinically preferred (inference): (i) needle survival and release fidelity under cyclic joint loading are untested; (ii) OARSI scoring, release kinetic characterization, and local-versus-systemic exposure partitioning are not standardized across studies; and (iii) no human OA MN efficacy data exist. Table 2 assembles the OA platforms along these comparative axes and marks explicitly the fabrication and pharmacokinetic fields that the primary literature must supply for a fully quantitative comparison.

A proposed minimum reporting checklist for MSK-MN physical characterization is given (Table 3). To make future musculoskeletal microneedle studies directly comparable and to close the reporting gap identified above, we recommend that primary reports state, at a minimum, the following physical characterization items (with the measurement method in each case) [68,74]:

### 4.3. Rheumatoid Arthritis and Joint Inflammation

RA is dominated systemically by methotrexate, JAK inhibitors, and biologics (anti-TNF, anti-IL-6R, and anti-CD20), all of which carry well-documented infection risks. Localized MN delivery can, in principle, decouple efficacy from systemic exposure [75,76,77]. Recent designs include a GelMA MN releasing a TNF-α/IL-6R dual-specific fenobody for sustained synovial cytokine blockade with concurrent bone-regenerative outcomes [16]; a fucoidan MN that reprograms macrophage polarization through ROS-responsive cargo release [46]; a PUMA-gene-and-celastrol synergy MN that restores synovial homeostasis [78]; an HA MN delivering naringin-loaded PEGylated terpesome that modulates TGF-β1 and oxidative stress [42]; a layered DMN that simultaneously addresses skin and joint lesions in psoriatic arthritis [73]; a GelMA-SilMA MN delivering DSPE-PEG2K-TAT-modified quercetin liposomes through the caspase-8/3/GSDME pathway [32]; a Phaseolus polysaccharide MN for sinomenine codelivery [29]; a self-enhancing Fenton-reaction nano-reactor MN for inflammatory microenvironment remodeling [63]; and a Janus sodium alginate/polyacrylamide/guar gum bilayer MN combining photodynamic and nitric oxide gas therapy for biofilm-complicated wounds, with translatable principles for septic arthritis [33,69,79].

A unifying design principle in the most successful RA MN platforms is the orchestrated reprogramming of the macrophage axis (M1 → M2) [16,46,78], which echoes the macrophage polarization framework now central to bone, cartilage, and IVD regeneration as well [17,19,31,80]. From a translational standpoint, transdermal MN delivery in RA is expected to be most attractive in three contexts: (i) home-administered maintenance dosing of biologic monotherapies between in-clinic visits, reducing the burden of injection site rotation and pharmacy distribution; (ii) localized “flare management” doses that decouple acute peri-articular cytokine spikes from systemic immunosuppression; and (iii) combination dosing of DMARDs with chondroprotective adjuvants in a single, layered MN patch. Nevertheless, the synovium’s extensive lymphatic drainage means that even peri-articular MNs deliver a non-negligible systemic dose of small-molecule payloads, and dose–response studies that explicitly partition local vs. systemic exposure are scarce in the published RA MN literature. These RA platforms are also seldom benchmarked against the clinical standard of systemic biologics, so whether localized MN delivery confers a genuine therapeutic index advantage over subcutaneous anti-TNF dosing remains an open and largely untested question [81,82].

### 4.4. Intervertebral Disc Degeneration

IVDD is the principal mechanistic driver of lower-back pain and a leading cause of MSK-related disability. The avascular nucleus pulposus (NP), with its hostile pro-inflammatory and acidic milieu, is notoriously refractory to local biologics: high-molecular-weight payloads injected through the annulus fibrosus (AF) leak through the iatrogenic puncture track and undergo rapid systemic clearance, while small molecules diffuse poorly through dense AF lamellae. Conventional minimally invasive percutaneous models (e.g., the rhesus-monkey early-degeneration model [56]) show that even a 22-gauge needle puncture itself accelerates degeneration, raising the bar for any AF-traversing device. Although the IVD is, therefore, anatomically the least accessible MSK target, four landmark 2023–2025 papers redefine what MN technology can do for IVDD. (i) A high-strength smart MN with “offensive and defensive” polymer architecture penetrates the annulus fibrosus without nucleus extrusion and locally releases anti-degenerative cargo [56]. (ii) A self-powered triboelectric MN releases optogenetically engineered EVs in response to the spine’s natural mechanical loading, providing a wholly autonomous closed-loop dosing platform [18]. (iii) Thread-structured MNs loaded with engineered exosomes restore mitophagy and ECM homeostasis in the annulus fibrosus, addressing one of the most refractory MSK regenerative medicine challenges [57]. (iv) A 2025 *Cell Reports Medicine* study demonstrated MN-mediated delivery of CAR-M-like engineered macrophages with enhanced efferocytosis capacity, alleviating IVDD in rodent models [17]. Together with self-healing hydrogels loaded with autophagy-promoting *Spatholobi caulis* extracts that complement MN delivery [83], these systems show that the synovial cartilage delivery paradigm is being successfully extended to the IVD.

Several practical considerations distinguish IVD MN designs from joint MN designs. First, the AF must be penetrated without creating a leak path that would itself accelerate degeneration; this implies smaller-diameter, sharper, and possibly self-sealing MN geometries—a niche where high-aspect-ratio 2PP-printed MNs and barbed thread MNs both offer advantages [56,57]. Second, the NP residence time of payloads is naturally extended by the disc’s compartmentalized anatomy, so even short-acting cargos can achieve clinically meaningful effects, but this also means that cargo distribution within the disc volume must be characterized by quantitative imaging (e.g., gadolinium-conjugated payload tracking) rather than gross histology alone. Third, intervention timing matters: most MN-IVDD studies use rodent models in which degeneration is induced by needle puncture and treated within days, whereas clinical IVDD evolves over years; longer-duration preclinical studies are needed before claims of regenerative reversal can be transferred to patient practice. A further unaddressed limitation is mechanical: the disc bears a continuous axial load, yet none of the IVDD MN studies report needle survival or matrix restoration durability under physiologic loading, leaving the central engineering challenge of this indication unresolved.

### 4.5. Tendon, Ligament, and Skeletal Muscle

The literature on tendon and skeletal muscle MN is younger than that on the bone, OA, and IVD subfields, but is accelerating rapidly. A carbonized wormwood photothermal MN patch promoted skeletal muscle repair through combined NIR-triggered hyperthermia and herbal extract release [58]. A bioresorbable electronic MN platform combined Mg electrode wireless electrotherapy with drug release for combined neuromuscular and metabolic indications [34]. A 2026 *Biomaterials* study demonstrated cardiac and skeletal-muscle delivery of biotherapeutics with a blood vessel epicardial-substance-targeting peptide, illustrating how MN platforms can be coupled to homing peptides for tissue specificity [84]. Beyond MNs, recent biomimetic skeletal muscle constructs engineered using induced myogenic progenitor cells [85] establish the cellular substrates that MN-based local cytokine delivery may eventually reinforce in vivo.

For tendon repair, the recombinant collagen MN platform for soft-tissue infection [45] points to the use of collagen-mimetic MNs as a tendon-compatible matrix, while the broader landscape of transdermal arthritic injury delivery has been comprehensively reviewed by Subramanian et al. [15].

The skeletal muscle compartment is particularly attractive for MN-based regenerative therapy because muscle damage occurs at predictable lesion locations (sport injuries, eccentric contractile damage, and post-surgical scarring) and because the satellite cell niche is amenable to local growth factor and EV stimulation. Combining an MN with a satellite-cell-supportive ECM mimetic—for example, a GelMA tip layer doped with insulin-like growth factor 1 (IGF-1) and a slowly degrading PLGA backbone delivering a TGF-β-pathway-modulating siRNA—would, in principle, recapitulate the cytokine cascade of physiological muscle regeneration [85], which already demonstrates that engineered myogenic constructs can self-renew and integrate by providing an in situ trophic micro-environment immediately around the construct. For tendinopathy, the principal opportunities are in the localized delivery of platelet-rich plasma derivatives, anti-inflammatory exosomes, and growth factors such as TGF-β1 and BMP-12, all of which currently rely on injection regimens that suffer from the same residence time problems as intra-articular injections. Critically, tendon and skeletal muscle MN work remains the least developed of the indication families surveyed here, with few controlled functional outcomes (force generation, gait, or tendon tensile recovery) and a near-total absence of comparative dosing studies, so conclusions in this space should be regarded as preliminary.

### 4.6. Periodontal, Peri-Implant, and Craniofacial MSK Applications

Although orofacial structures are not always grouped with orthopedic MSK indications, the underlying biology of alveolar bone, periodontal ligament, and temporomandibular cartilage shares a great deal of mechanism, and the literature on polymer MNs in these tissues is correspondingly informative. Buccal MNs coated with optimized hydrogels containing naproxen and dexamethasone have been characterized for transmucosal anti-inflammatory delivery [59], and a comprehensive 2025 review of biological-macromolecule-based periodontal regeneration platforms documents the rapid integration of HA, chitosan, alginate, silk fibroin, hydroxyapatite, β-TCP, and bioactive glass into orofacial regenerative biomaterials [60], chemistries that overlap heavily with the polymer MN palette and that already have FDA clearances in dental products. The orofacial setting is, therefore, a useful clinical stage proving ground for MN-MSK technologies before they migrate to deeper, harder-to-monitor MSK targets. Even so, the orofacial evidence is still largely proof-of-concept and is reported as isolated demonstrations rather than as a comparable series, which limits the strength of cross-study inference even in this more accessible compartment.

### 4.7. Cross-Cutting Principles from the Indication Map

Across the six indication families surveyed [82] in Section 4.1, Section 4.2, Section 4.3, Section 4.4, Section 4.5 and Section 4.6, three cross-cutting design principles emerge. (i) Layered architecture for staged release: Indications with biphasic pathology (OA’s anti-inflammatory + chondroprotective phases; bone non-union’s chemotaxis + osteoinduction phases; and IVDD’s mechanical-protection + matrix-restoration phases) consistently benefit from layered MNs that decouple early and late kinetics, and the most successful platforms (denosumab DMN [19], lubricating multi-stage MN [36], thread-structured EV MN [57], and TNF-α/IL-6R fenobody GelMA MN [16]) all exhibit this design feature. (ii) Endogenous trigger exploitation: The MSK micro-environment offers ROS, pH, and mechanical-loading triggers that match the indication-specific pathology, and platforms that explicitly exploit these triggers (Fenton-reactor RA MN [63], triboelectric IVD MN [18], and ROS-responsive fucoidan MN [46]) tend to outperform constitutively releasing comparators. (iii) Cell + biologic + biomaterial co-design: The most translationally promising systems are those in which the MN polymer matrix is itself bioactive (GelMA chondrogenic priming, silk fibroin antimicrobial activity, and HA lubricating function), the cargo is a cell or EV that carries multi-modal regenerative cues, and the architecture explicitly matches the tissue mechanics, exemplified by the GelMA-SilMA quercetin liposome RA MN [32] and the silk-fibroin BMSC-EV mucosal MN [20]. These principles can be transposed back into scaffold engineering as well; for example, the chondrogenic-priming bioceramic logic [22] could be rendered into MN tip layers and the staged growth factor release strategy of double cryogels [4] into multi-layer DMN backings (these scaffold-to-MN transpositions are forward-looking proposals by the authors, not yet experimentally demonstrated). One methodological weakness cuts across all three principles: outcomes are reported descriptively and against non-uniform models and timescales, fabrication parameters (needle geometry, density, insertion force, and mechanical strength) are rarely linked quantitatively to therapeutic outcome, and failure modes are seldom disclosed, so the field’s comparative claims currently rest on narrative synthesis rather than on standardized, head-to-head evidence.

## 5. Smart and Stimuli-Responsive MN Systems

**Figure 4:**
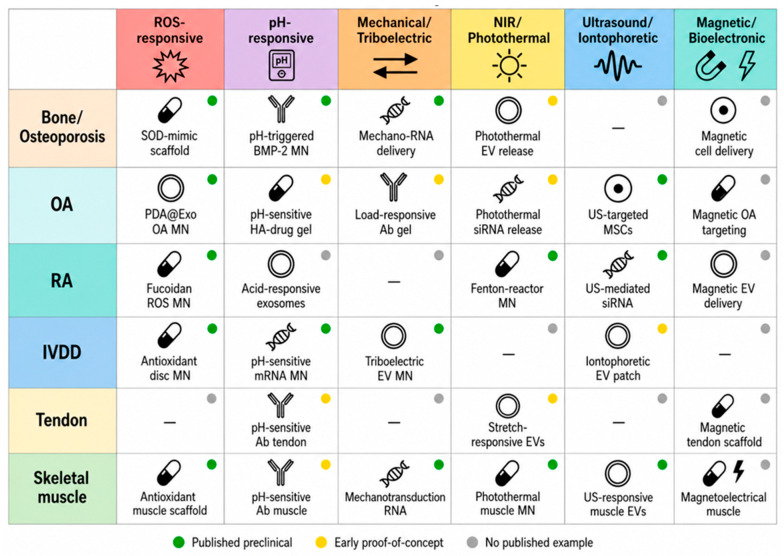
Stimulus trigger × MSK indication matrix for smart, stimuli-responsive polymer microneedles. Rows enumerate the six principal MSK disease families (bone/osteoporosis, osteoarthritis, rheumatoid arthritis, intervertebral disc degeneration, tendon, and skeletal muscle). Columns enumerate six endogenous or exogenous trigger classes (reactive oxygen species, pH, mechanical/triboelectric, near-infrared/photothermal, ultrasound/iontophoretic, and magnetic/bioelectronic). Cells are populated with the cargo class (small molecule, biologic, nucleic acid, EV, or cell) and an evidence strength indicator (green = published preclinical data; yellow = early proof-of-concept; grey = no published example), summarizing the 2018–2026 literature. Representative exemplars include ROS-responsive fucoidan MN for RA [43], triboelectric MN for IVDD EV delivery [18], photothermal MN for skeletal muscle [58], and Fenton-reactor MN for RA [62]. Abbreviations: EV, extracellular vesicle; IVDD, intervertebral disc degeneration; MN, microneedle; NIR, near-infrared; OA, osteoarthritis; RA, rheumatoid arthritis; ROS, reactive oxygen species.

The MSK micro-environment offers multiple endogenous and exogenous triggers that can gate MN release. We organize emerging “smart” MN designs by trigger class (Figure 4).

### 5.1. ROS-, pH-, and Glucose-Responsive Systems

OA, RA, IVDD, and infected bone defects are characterized by elevated ROS and acidic pH. Boron-based polymers exploit the boronate–diol equilibrium for ROS- and pH-dual responsiveness [53]. Smart MNs with porous polymer coatings achieve pH-responsive drug delivery without requiring complex chemistry [48]. Glucose-responsive silk-fibroin smart MNs combining a semi-IPN hydrogel with glucose-oxidase-mediated insulin release [44,86] illustrate the broader principle, transferable to MSK indications where local glycemic control around diabetic non-unions is desirable.

### 5.2. Mechanical-, Ultrasound-, and Triboelectric-Responsive Systems

The mechanically active nature of the MSK system makes it ideally suited to MNs that release on physical stimulation. The triboelectric IVD MN [18] generates voltage from natural spinal loading and transduces this into optogenetic EV release. Ultrasonically and iontophoretically enhanced DMN patches improve transdermal flux of high-molecular-weight cargos relevant to peri-tendon and peri-articular delivery [87]. A microcurrent-integrated MN system enhances PTH delivery by iontophoresis [66]. These external trigger systems offer the additional advantage of patient- or clinician-controlled dosing.

### 5.3. Thermal, Photothermal, and Photodynamic Systems

Carbonized photothermal patches for skeletal muscle damage [58] and Janus photodynamic-NO-releasing MNs [33] represent the convergence of MN platforms with photothermal/photodynamic therapy. NIR-responsive systems benefit from the relatively transparent skin window in the 700–1000 nm range and have been combined with self-enhancing Fenton chemistry for RA [63].

### 5.4. Magnetic, Electric, and Bioelectronic MNs

Bioresorbable electronic MNs based on Mo electrodes and Mg interconnects deliver pulsed electric stimulation alongside drug release, providing combined regenerative and biophysical cues [34]. The integration of MN arrays with flexible electronics is poised to enable wireless, implantable MSK regenerative platforms in which electrical stimulation, real-time biosensing, and on-demand drug release are coordinated by an embedded controller. Such platforms could close the loop between disease state sensing (e.g., pH, glucose, IL-6, and ROS) and therapeutic dosing, an architecture that is conceptually compatible with the closed-loop logic of the triboelectric IVD MN [18].

Magnetic-responsive microneedles: status and design barriers. In contrast with the mechanical-, ROS- and photothermal-responsive systems above, no magnetic-field-responsive polymer microneedle has yet been reported for a musculoskeletal indication; the magnetic/bioelectronic column of Figure 4 is, therefore, populated only by the electric/bioelectronic class. Magneto-responsive microneedles have been demonstrated in other settings—for example, magnetically steered microneedle “robots” that orient and insert into the intestinal wall for macromolecule delivery [88] and the magnet-responsive class catalogued in recent stimuli-responsive MN reviews [89]—so the concept is feasible in principle. Several design barriers, however, specifically discourage its musculoskeletal translation (authors’ analysis): (i) magnetic actuation requires embedding ferro-/super-paramagnetic particles (e.g., iron oxide) in the polymer matrix, which complicates biodegradation, raises long-term retention and biocompatibility questions in load-bearing tissue, and creates MRI susceptibility artefacts that would obscure the very joints and discs being monitored; (ii) generating useful force or triggered release at the 2–10 mm depth of musculoskeletal targets needs strong external field gradients and bulky hardware, which negates the patient-administrable, self-applied advantage that motivates microneedle use; (iii) the added magnetic component increases manufacturing and regulatory complexity (a combination drug–device–nanomaterial product) relative to dissolving or hydrogel MNs that already meet musculoskeletal needs; and (iv) the endogenous triggers most relevant to musculoskeletal pathology—mechanical loading, ROS and pH—are better matched by the mechanically and chemically responsive platforms already discussed. We, therefore, identify magnetic-responsive MSK microneedles as a conceptually open but, at present, low-priority design space.

### 5.5. Smart MNs for Cell, EV, and Gene Delivery

The therapeutic frontier for MSK regeneration is increasingly cellular and biologic. Microneedle-mediated EV delivery has now been formally reviewed [80], and indication-specific systems include MSC-EV MNs that drive macrophage polarization [90] and immunomodulatory exosome platforms with biomaterial-driven delivery [91]. Gene therapy MNs cover both transdermal siRNA/mRNA delivery [92] and in-MSK applications such as the PUMA gene celastrol synergy MN for RA [78]. Engineered EVs co-delivered through thread-structured MNs for annulus fibrosus repair [57] and through triboelectric IVD MNs [18] showcase the convergence of EV bioengineering with MN-based localized administration.

A core–shell MN system for stable fibroblast delivery in cell-based therapies [64] anticipates the eventual MN-mediated delivery of MSCs, induced myogenic progenitor cells [85], or chondroprogenitor populations for MSK regeneration. Hydrogel-loaded EVs more broadly have been comprehensively reviewed in the context of tissue repair [93,94], with the convergence of EV bioengineering and MN-format depots emerging as one of the highest-velocity subfields of regenerative pharmacology in 2024–2026. Sericin-/HA-methacrylate composite MNs loaded with EVs for diabetic wounds [90] illustrate how natural-protein chemistries can be tuned to release vesicular cargos under controlled enzymatic degradation kinetics, with direct relevance to chronic MSK wounds such as non-healing ulcers overlying osteomyelitic bone.

A complementary trend is the design of “self-recruiting” MN systems that release chemoattractants to draw endogenous repair cells (MSCs, monocytes, and satellite cells) into the lesion rather than delivering exogenous cells. The SDF-1/BMP-2 sequential cryogel paradigm [4] and the heparin-functionalized injectable cryogels for neovascularization [23] established the mechanistic blueprint for chemoattractant-then-effector dosing in scaffold form; transposing this rationale into MN platforms is the next logical step.

## 6. Conclusions and Future Perspective

Polymer microneedles (MNs) have matured from a transdermal vaccine niche into a rich, MSK-specific drug delivery platform that can localize small molecules, biologics, nucleic acids, extracellular vesicles (EVs), and even cells with millimeter-scale precision and patient-administrable simplicity. The 2018–2026 literature shows a clear trajectory: coated-needle PTH for osteoporosis [25]; dissolving and hydrogel MNs for OA and RA [19,31,32,36,46]; high-strength smart and bioelectronic MNs that now reach the previously inaccessible intervertebral disc and loaded peri-articular niches [17,18,34,56,57]; and photothermal or bioresorbable-electronic MNs for skeletal muscle [58,84]. Polymer chemistry has co-evolved: PLGA dominates sustained-release designs [24,38]; HA, GelMA, silk fibroin, and chitosan increasingly provide bioactive matrices that recapitulate elements of the native ECM [16,20,32,43,44,45]; and composite MNs combine bioactive ceramics with stimulus-responsive polymers to deliver multi-modal cues matching the inflammation–regeneration sequence of MSK healing [31,53,63].

Despite this momentum, several gaps will shape the next phase of the field. First, few MNs are engineered to tolerate the cyclic mechanical loading of bone, cartilage, tendon, and IVD; transposing load-bearing scaffold characterization [1,3,7,47,55] into MN evaluation is overdue. Second, sustained dosing across MSK healing timescales (weeks to months) remains difficult. PLGA-based and lyophilized composite platforms have extended release to 2–4 weeks [24,35,38], but pulsed or refillable designs are needed, and the double-cryogel sequential-release logic [4] offers a transferable blueprint. Third, cell- and EV-loaded MNs still face joint challenges of dehydration, shear during insertion, and polymer-curing toxicity; core–shell hydrogel designs and mild crosslinking chemistries [43,64] offer a path, but standardized post-insertion viability assays are absent. Fourth, stimuli-responsive MNs need systematic toxicology of degradation products (boronate esters, photothermal carbon, and iron-Fenton catalysts) and stability data over clinically relevant storage [53,63]. Fifth, vascularization of deep MSK targets cannot be solved by MNs alone; integration with heparin-functionalized cryogels [23] and VEGF-overexpressed MSC platforms [49] is required. Finally, inflammation–regeneration co-design—exemplified by the TNF-α/IL-6R fenobody MN [16], macrophage-reprogramming fucoidan MN [46], and CAR-M MN for IVDD [17]—will increasingly displace single-modality platforms. A sober translational caveat must accompany this optimism: with the sole exception of the transdermal PTH and abaloparatide osteoporosis systems—which have reached phase 1–3 trials [95,96]—clinical efficacy data for musculoskeletal microneedles do not yet exist, and the evidence base remains overwhelmingly preclinical and rodent-dominated [97]; clinician- and patient-administered first-in-human dermal safety and dose-finding studies (such as the registered denosumab microneedle trial) are the necessary next step before any of the platforms reviewed here can be considered clinically established.

The most promising near-term opportunity is the hybrid MN–scaffold combination product, pairing the patient-administrable, dose-titratable advantages of MNs with the load-bearing and vascularization-supporting advantages of macroporous and 3D-printed scaffolds [3,6,21,61,85,98]. Although bone and osteoporosis are the most immediate beneficiaries—because load-bearing repair most needs the mechanical support that macroporous and 3D-printed scaffolds provide [70,81]—this hybrid MN–scaffold logic is not confined to bone; it extends across the musculoskeletal spectrum, including osteochondral and cartilage repair (a chondroprotective or chondrogenic-priming MN tip mounted on a calcified-cartilage-supporting scaffold), intervertebral disc treatment (an annulus-sealing microneedle integrated with a nucleus-supporting hydrogel), and tendon–bone interface reconstruction. Cross-disciplinary integration of biomaterial engineering, micro-fabrication [99], immunology, and clinical orthopedics—together with rigorous standardized testing and transparent reporting of failure modes—will determine whether polymer-MN-based MSK delivery moves from a fast-growing publication trend into clinical practice over the next decade [100].

## Figures and Tables

**Table 1:** At-a-glance summary of polymer-microneedle platforms across the musculoskeletal disease spectrum.

MSK Indication	Polymer Chemistries	Architectures	Representative Payloads	Outcome Theme	Refs
Osteoporosis	Ti/polymer, HA, HA/CMC (+/− microcurrent)	Coated solid, DMN	Teriparatide/PTH(1-34)	Systemic PK/osteoanabolic	[25,26,27,65]
Osteoarthritis	HA, PLGA, PVP/PEG, polydopamine-PEGDA	DMN, hydrogel MN, lubricating MN	Denosumab, MSC-exosomes, KGN + DEX, triptolide, celecoxib	Chondroprotection/anti-inflammatory	[19,30,35,40,61,67]
Rheumatoid arthritis	GelMA, GelMA-SilMA, fucoidan, HA	Hydrogel MN, DMN	TNF-a/IL-6R fenobody, quercetin liposome, naringin, M2 cargo	Macrophage reprogramming/cytokine blockade	[16,31,41,45]
Psoriatic arthritis	HA (layered)	Layered DMN	Methotrexate + topical	Concurrent skin + joint relief	[68]
Intervertebral disc degeneration	High-strength/triboelectric/thread polymers	Smart composite and electronic MN, thread-structured MN	Anti-degenerative cargo, optogenetic EVs, exosomes, CAR-M	AF penetration + disc regeneration	[17,18,55,56]
Skeletal muscle	Carbonized polymer, Mg/Mo bioresorbable electronics	Photothermal MN, bioresorbable electronic MN	Herbal + NIR, drug + electrotherapy	Muscle repair/wireless dosing	[33,57]
Periodontal/orofacial	HA coated	Buccal coated MN	Naproxen + DEX	Buccal anti-inflammatory	[58]
Tendon (infection)	Recombinant collagen	DMN	Cu-DNA nanoparticles	Antibacterial	[44]

**Table 2:** Comparative appraisal of representative osteoarthritis (OA) microneedle platforms in terms of fabrication, release, model, outcome, and limitation axes.

Platform (Design Class)	Polymer/Architecture	Release Strategy and Duration	Model and Follow-Up	Reported Outcome	Key Limitation/Data Gap	Reference
Lubricating multi-stage MN	Surface-engineered lubricating MN; multi-stage	Lubricant burst + sustained anti-inflammatory; duration not reported	Rat MIA; 8 weeks	Significant OARSI score reduction over 8 weeks	Geometry/strength not reported; rodent only; no loading durability data	[35]
Denosumab DMN	Dissolving MN	Tip-dissolution bolus; duration not reported	Canine + rodent OA	Targets macrophage senescence; principal large-animal dataset ↓ synovial inflammation, cartilage erosion and pain; efficacy comparable to intra-articular injection while avoiding systemic exposure (single-cell RNA-seq: fewer senescent synovial macrophages) [19]	Effect size/PK not reported; release not controlled	[19]
PDA-exosome (PDA@Exo) MN	ROS-scavenging matrix; polydopamine-coated MSC exosomes	ROS-responsive release	Rodent OA	Chondroprotection via PI3K-Akt-mTOR; rebalances anabolic/catabolic	Geometry/dose/follow-up not reported; rodent only	[30]
Liposome-triptolide DMN	Dissolving MN; liposome loaded	Bolus via tip dissolution	Intra-articular; model not reported	Improved IA delivery; reduced systemic toxicity ↓ knee swelling and TNF-α/IL-1β/IL-6; ↓ cartilage destruction by micro-CT/histology (rat OA; ~200 µm penetration) [63]	Quantitative outcome/follow-up not reported	[61]
Polysaccharide MN (C. frondosa + 3-acetylaconitine)	Polysaccharide MN	Bolus; duration not reported	Rodent; model not reported	Combined analgesic/anti-inflammatory/chondroprotective	Effect size/geometry not reported	[66]
GAG/CS/HA PVP-PEG MN	PVP-PEG MN; ECM molecule cargo	Dissolution release	Not reported	Demonstrates ECM-constituent delivery via MN	Efficacy endpoints/model not reported	[40]
Microemulsion celecoxib + alpha-LA DMN	Dissolving MN; microemulsion incorporated	Bolus solubilized codelivery	Not reported	Carries hydrophobic NSAID combination for IA use	Outcome/PK not reported	[67]
Human placental-tissue MN	Polymeric dissolving MN	Tip-dissolution delivery of processed human placental tissue	Surgical OA model (rodent)	Dual chondroprotective + immunomodulatory effects	Quantitative geometry/PK not reported	[39]

Note: “Not reported” indicates the parameter was not stated in the primary publication, not that the platform lacks the property. The standardized reporting checklist proposed in Section In-Depth Comparison of OA Microneedle Platforms: Fabrication, Design Logic, and Outcomes is intended to close these gaps in future MSK-MN studies.

**Table 3:** Recommended minimum physical-characterization reporting checklist for musculoskeletal microneedle studies.

Parameter	Minimum Item to Report (With Method)
Needle length/height	Mean ± SD height in µm; measurement method (optical/SEM)
Tip radius/sharpness	Tip radius in µm; imaging method
Base diameter and wall (hollow)	Base diameter in µm; lumen/wall dimensions for hollow MNs
Aspect ratio	Height-to-base ratio (dimensionless)
Array pitch/tip density	Center-to-center spacing (µm) and needles cm^−2^
Needle count/patch area	Number of needles and total patch area
Insertion force and method	Force per needle/array (N); test rig and substrate
Mechanical strength (failure/compression)	Failure or 10–30% compression force (N/needle); texture analyzer/UTM method
Penetration depth and model	Depth in µm; model used (parafilm-M, ex vivo skin, or target tissue)
Dissolution/degradation	Time to dissolve or degrade in situ; medium and assay
Drug loading and release	Loading (µg/patch); in vitro/in vivo release method and duration

## Data Availability

No new data were created or analyzed in this study. Data sharing is not applicable to this article.

## Abbreviations

**Abbreviation**	**Definition**
2PP	Two-photon polymerization
3D	Three-dimensional
AF	Annulus fibrosus
ALA	Alpha-linolenic acid
AUC	Area under the curve
BMD	Bone mineral density
BMP-2	Bone morphogenetic protein 2
BMSC	Bone marrow mesenchymal stem cell
beta-TCP	Beta-tricalcium phosphate
CAR-M	Chimeric antigen receptor macrophage
Cmax	Maximum concentration
CMC	Carboxymethyl cellulose
CS	Chondroitin sulfate
DEX	Dexamethasone
DLP	Digital light processing
DMARD	Disease-modifying antirheumatic drug
DMN	Dissolving microneedle
DMOAD	Disease-modifying osteoarthritis drug
ECM	Extracellular matrix
EV	Extracellular vesicle
GAG	Glycosaminoglycan
GelMA	Gelatin methacryloyl
GSDME	Gasdermin E
HA	Hyaluronic acid
HMN	Hydrogel-forming microneedle
IGF-1	Insulin-like growth factor 1
IL	Interleukin
ISF	Interstitial fluid
IVD	Intervertebral disc
IVDD	Intervertebral disc degeneration
JAK	Janus kinase
KGN	Kartogenin
LPS	Lipopolysaccharide
MIA	Monosodium iodoacetate
MMP	Matrix metalloproteinase
MN	Microneedle
MSC	Mesenchymal stem cell
MSK	Musculoskeletal
NIR	Near-infrared
NP	Nucleus pulposus
NSAID	Non-steroidal anti-inflammatory drug
OA	Osteoarthritis
OARSI	Osteoarthritis Research Society International
PCL	Poly(epsilon-caprolactone)
PDA	Polydopamine
PEG	Poly(ethylene glycol)
PEGDA	Poly(ethylene glycol) diacrylate
PK	Pharmacokinetic
PLA	Poly(lactic acid)
PLGA	Poly(lactic-co-glycolic acid)
PRP	Platelet-rich plasma
PTH	Parathyroid hormone
PUMA	p53-upregulated modulator of apoptosis
PVA	Poly(vinyl alcohol)
PVP	Poly(vinyl pyrrolidone)
RA	Rheumatoid arthritis
ROS	Reactive oxygen species
SDF-1	Stromal cell-derived factor 1
SilMA	Silk fibroin methacryloyl
siRNA	Small interfering RNA
TGF-beta	Transforming growth factor beta
TNF-alpha	Tumor necrosis factor alpha
TPMS	Triply periodic minimal surface
VEGF	Vascular endothelial growth factor
